# Oral Administration of the Japanese Traditional Medicine Keishibukuryogan-ka-yokuinin Decreases Reactive Oxygen Metabolites in Rat Plasma: Identification of Chemical Constituents Contributing to Antioxidant Activity

**DOI:** 10.3390/molecules22020256

**Published:** 2017-02-08

**Authors:** Yosuke Matsubara, Takashi Matsumoto, Kyoji Sekiguchi, Junichi Koseki, Atsushi Kaneko, Takuji Yamaguchi, Yumiko Kurihara, Hiroyuki Kobayashi

**Affiliations:** 1 Tsumura Research Laboratories, Tsumura & Co., Ibaraki 300-1192, Japan; matsumoto_takashi@mail.tsumura.co.jp (T.M.); sekiguchi_kyouji@mail.tsumura.co.jp (K.S.); koseki_junichi@mail.tsumura.co.jp (J.K.); kaneko_atsushi@mail.tsumura.co.jp (A.K.); yamaguchi_takuji@mail.tsumura.co.jp (T.Y.); 2 Center for Advanced Kampo Medicine and Clinical Research, Juntendo Graduate School of Medicine, Tokyo 113-8421, Japan; yu-kurihara@juntendo.ac.jp (Y.K.); koba@juntendo.ac.jp (H.K.); 3 Department of Hospital Administration, Juntendo University Graduate School of Medicine, Tokyo 113-8421, Japan

**Keywords:** antioxidant, gallic acid, Keishibukuryogan-ka-yokuinin, lignan, plasma pharmacokinetics, reactive oxygen metabolites, reactive oxygen species

## Abstract

Insufficient detoxification and/or overproduction of reactive oxygen species (ROS) induce cellular and tissue damage, and generated reactive oxygen metabolites become exacerbating factors of dermatitis. Keishibukuryogan-ka-yokuinin (KBGY) is a traditional Japanese medicine prescribed to treat dermatitis such as acne vulgaris. Our aim was to verify the antioxidant properties of KBGY, and identify its active constituents by blood pharmacokinetic techniques. Chemical constituents were quantified in extracts of KBGY, crude components, and the plasma of rats treated with a single oral administration of KBGY. Twenty-three KBGY compounds were detected in plasma, including gallic acid, prunasin, paeoniflorin, and azelaic acid, which have been reported to be effective for inflammation. KBGY decreased level of the diacron-reactive oxygen metabolites (d-ROMs) in plasma. ROS-scavenging and lipid hydroperoxide (LPO) generation assays revealed that gallic acid, 3-*O*-methylgallic acid, (+)-catechin, and lariciresinol possess strong antioxidant activities. Gallic acid was active at a similar concentration to the maximum plasma concentration, therefore, our findings indicate that gallic acid is an important active constituent contributing to the antioxidant effects of KBGY. KBGY and its active constituents may improve redox imbalances induced by oxidative stress as an optional treatment for skin diseases.

## 1. Introduction

Skin is chronically exposed to endogenous and environmental stimuli, leading to the harmful generation of reactive oxygen species (ROS) [1,2]. A growing body of evidence indicates that oxidative stress is involved in the damage of cellular constituents such as DNA, lipids, and proteins, as well as the release of pro-inflammatory mediators. Under normal physiological conditions, ROS level is under control; however, insufficient detoxification and/or overproduction of ROS generate oxidative stress, resulting in cellular and tissue damage. In the case of chronic inflammation, the antioxidant systems may be depleted and prolonged oxidative stress may occur [3]. Increased ROS and oxidation products are seen in many dermatologic disorders, including atopic dermatitis, psoriasis, vitiligo, pemphigus vulgaris and alopecia areata [4,5,6,7,8,9]. In patients with acute exacerbation of atopic dermatitis, 8-hydroxy-deoxyguanosine (a marker of oxidative DNA damage), acrolein-lysine adducts (markers of lipid peroxidation), and bilirubin oxidative metabolites (markers of bilirubin oxidative metabolism) are demonstrated to be increased significantly in urine and return to normal levels during disease remission [8,9]. To examine oxidative stress in patients with inflammatory diseases, several clinicians perform the diacron-reactive oxygen metabolites (d-ROMs) test which essentially determines the concentration of hydroperoxides (ROOH) in the blood, and proved to be a reliable and effective tool for readily evaluating oxidative stress in both humans and animals. Moreover, 13*S*-hydroperoxy-9*Z*,11*E*-octadecadienoic acid (13-HpODE), a representative lipid hydroperoxide produced by the oxidation of linoleic acid has been reported to inhibit the production of nitric oxide (NO) [10]. Because NO produced by vascular endothelial cells plays an important part on the regulation of vascular homeostasis, insufficient detoxification and/or overproduction of ROS lead to dysregulation of blood flow like ischemia. Some of the reactive oxygen metabolites can circulate in the blood due to their relative stability compared to ROS, and can become exacerbating factors of systemic and/or prolonged inflammation. Therefore, it is of importance in diagnosis and treatment for refractory dermatosis to understand the mutual relationship among peripheral blood flow, reactive oxygen products, and inflammation.

Keishibukuryogan-ka-yokuinin (KBGY) is a pharmaceutical-grade traditional Japanese medicine (kampo) that has been used widely for the treatment of skin conditions such as acne vulgaris, chapped skin, and freckles. KBGY is a combination drug consisting of Coicis Semen and another kampo medicine, Keishibukuryogan (KBG, gui-zhi-fu-ling-wan in Chinese), which contains the following five crude drugs; Cinnamomi Cortex, Paeoniae Radix, Persicae Semen, Poria, and Moutan Cortex. In Japan, gynecologists, dermatologists, and other clinicians prescribe KBG to treat symptoms of peripheral ischemia such as cold extremities [11]. KBG has been reported to decrease the level of reactive oxygen metabolites in rats [12], and to improve microvascular blood flow in humans [13,14]. On the other hand, Coicis Semen is prescribed in Japan for treating wart, chapped skin, acne vulgaris, and the other skin troubles, and the inhibitory effects on oxidation and inflammation have been reported [15]. However, the active constituents of KBG and/or KBGY and their pharmacokinetic profiles remain unclear. In the present study, we focused on the antioxidant potential of KBGY for decreasing reactive oxygen metabolites related to the pathogenesis and progression of skin diseases. We examined the blood pharmacokinetics of constituents of KBGY and their antioxidant effects using both in vivo and in vitro assays, to identify active constituents absorbed to the systemic circulation that contribute as key players in the antioxidant activity of KBGY.

## 2. Results and Discussion

### 2.1. Amounts of Chemical Constituents in the Extracts of KBGY and Component Crude Drugs

According to previous reports [16,17,18,19,20,21,22] and our research direction, 48 candidate compounds were selected; amounts of each candidate compound in the KBGY extract are represented by chemical class in Table 1.

This study revealed that 39 constituents were identified in KBGY. The content of paeoniflorin in the KBGY extract was the highest (17,000 μg/g) of all of compounds examined. Albiflorin, tetragalloyl glucose, pentagalloyl glucose, gallic acid, amygdalin, prunasin, and cinnamaldehyde were also major constituents (>1000 μg/g).

**Figure 1:**
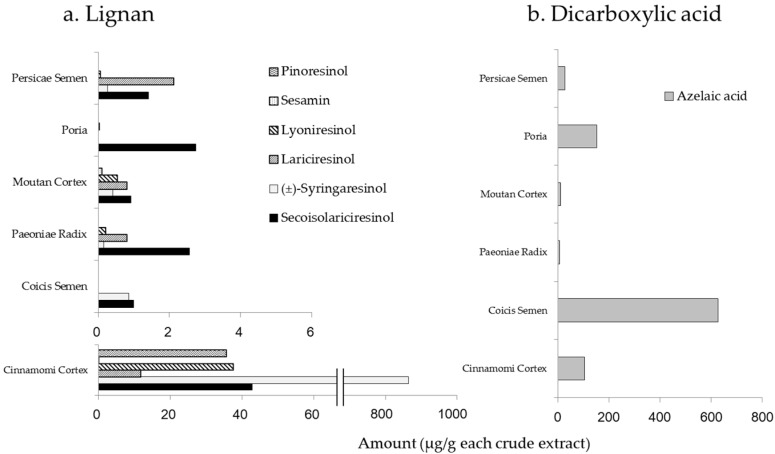
Identification of crude drugs containing lignans and azelaic acid. The amounts of lignans (**a**) and a dicarboxylic acid; azelaic acid (**b**) were measured by LC/MS/MS or GC/MS. The dry-powdered extracts of Cinnamomi Cortex, Paeoniae Radix, Persicae Semen, Poria, Moutan Cortex, and Coicis Semen were subjected to LC/MS/MS or GC/MS analyses to measure amounts of the following six lignans and azelaic acid; secoisolariciresinol, (±)-syringaresinol, lariciresinol, lyoniresinol, sesamin and pinoresinol. Data are shown as single results.

Lignans and dicarboxylic acids are contained in most plants, thus we examined the representative compounds in every component crude drug. Lignans were the most abundant in the extract of Cinnamoni Cortex, with (±)-syringaresinol particularly prominent (Figure 1a). Azelaic acid is used widely for the treatment of dermatitis such as rosacea and acne vulgaris [23,24], thus we determined its amounts in the component crude drugs. Our results demonstrated that the highest concentration of azelaic acid was found in the extract of Coicis Semen (Figure 1b).

Coicis Semen is an extract of the coix seed and belongs to the same family of Poaceae as wheat. Considering that azelaic acid is found in wheat and provided to manufacture pharmaceutical medications for skin conditions, we hypothesized that Coicis Semen must contain azelaic acid. Indeed, our result exhibited that Coicis Semen contained azelaic acid more sufficiently than the other crude drugs. Adipic acid was also quantified in Coicis Semen; however, the content was lower than that of azelaic acid, and there were no remarkable differences among the crude drugs (data not shown).

### 2.2. Identification and Quantification of Blood-Absorbed Constituents and Their Metabolites

Plasma kinetics of 23 compounds derived from KBGY was determined in rats after a single oral administration of KBGY (2 g/kg). As shown in Table 2, the most abundant constituent, paeoniflorin, was quantified at a *C*_max_ of 80.2 ng/mL 0.5 h after administration. Among the detected compounds, the plasma concentration of prunasin was the highest with a *C*_max_ of 1450 ng/mL 1 h after administration. Gallic acid was quantified at a *C*_max_ of 84.9 ng/mL 0.5 h after administration. The methylation metabolites of gallic acid, 3-*O*-methylgallic acid, and 4-*O*-methylgallic acid, were also detected in the plasma with *C*_max_ values of 12.7 and 159 ng/mL respectively, and the same *t*_max_ (1 h). The *C*_max_ of (+)-catechin was 22.6 ng/mL 2 h after administration. The most abundant lignan, (±)-syringaresinol, was quantified at a *C*_max_ of 0.213 ng/mL, while lariciresinol was identified in the highest concentration (1.68 ng/mL) among the lignans examined in this study.

**Figure 2:**
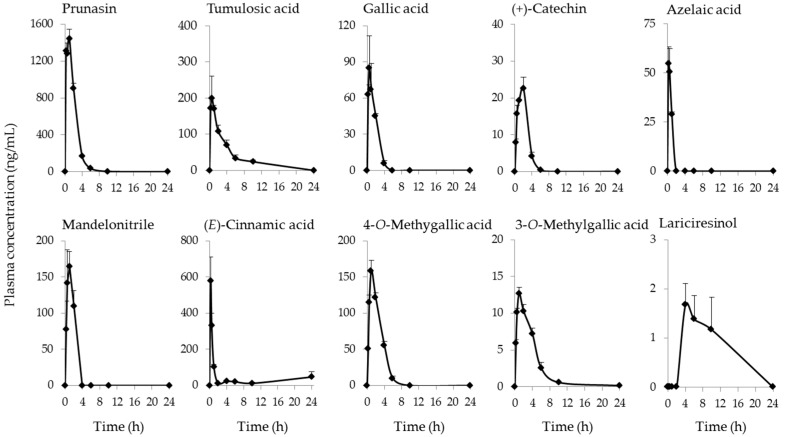
Time-courses of representative constituents in the plasma of KBGY-treated rats. Plasma samples were obtained at 0.25, 0.5, 1, 2, 4, 6, 10 and 24 h after single administration of KBGY (2 g/10 mL/kg, p.o.). Prunasin, tumulosic acid, gallic acid, (+)-catechin, mandelonitrile, (*E*)-cinnamic acid, 4-*O*-methylgallic acid, 3-*O*-methylgallic acid, azelaic acid, and lariciresinol in the plasma were measured by LC/MS/MS or GC/MS. Each data point represents the mean ± S.E. of triplicate results.

Figure 2 illustrates the plasma kinetics of the representative constituents and metabolites derived from KBGY. The present study is the first to clarify the plasma pharmacokinetic profiles of some KBGY constituents and their metabolites, although there are papers showing the profiles of representative constituents in the study of KBG [25,26,27]. The constituent with the highest concentration was prunasin, which is derived from Persicae Semen [22], and demonstrated a *C*_max_ of 1450 ng/mL 1 h after administration. The prunasin analogues, amygdalin and manderonitrile, were also quantified in plasma at high concentrations. Prunasin has been demonstrated to inhibit the production of pro-inflammatory cytokines by human keratinocytes [28], implying a possible anti-inflammatory action. The following compounds were also identified in plasma at relatively-high concentrations; tumulosic acid, paeoniflorin, 4-*O*-methylgallic acid, gallic acid, manderonitrile, and (*E*)-cinnamic acid. These constituents have been reported to have various pharmacological effects in cell culture systems and animal models focusing on dermatitis and blood flow [17,18,29,30,31,32,33,34,35,36].

Azelaic acid was also identified in plasma of rats treated with KBGY. Azelaic acid gel is used to clear the bumps, lesions, and swelling caused by rosacea, and is also prescribed to treat acne vulgaris [23,24]. There are several papers showing effects of azelaic acid on the systemic and local (skin) inflammation in vivo and in vitro [37,38,39]. Azelaic acid also suppresses the production of ROS such as the superoxide anion and hydroxyl radicals by human neutrophils, although it reportedly did not influence ROS generation in a cell-free system [40]. These findings provide a possible mechanism by which azelaic acid may exhibit antioxidant effects via the suppression of ROS-generating enzymatic reactions such as nicotinamide adenine dinucleotide phosphate-oxidase (NADPH oxidase), but not by scavenging ROS [41].

### 2.3. Change in Oxidative Stress Parameter by KBGY Administration

**Figure 3:**
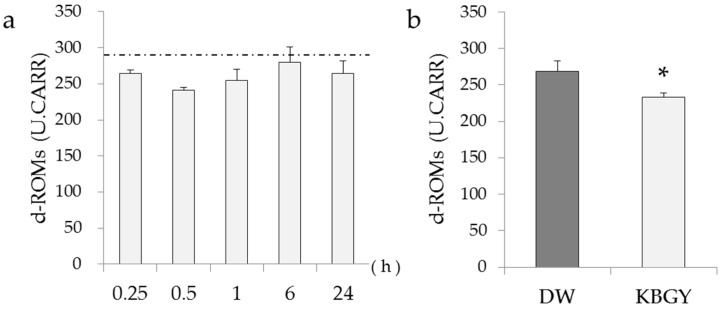
Decreased level of reactive oxygen metabolites by administration of KBGY. Plasma samples were obtained at 0.25, 0.5, 1, 2, 4, 6, 10 and 24 h after single administration of KBGY (2 g/10 mL/kg, p.o.). The levels of diacron-reactive oxygen metabolites (d-ROMs) in plasma were evaluated. (**a**) Time-course was investigated (*n* = 3). The levels of d-ROMs of the plasma obtained from non-administrated rats were represented as dashed lines; (**b**) Plasma samples were obtained 0.5 h after the administration of KBGY or distilled water (DW). Data are shown as the mean ± S.E. of 10 rats. * *p* < 0.05 indicates significant versus DW group from the unpaired Student’s *t*-test.

The levels of d-ROMs in the plasma samples used for plasma pharmacokinetic studies were measured preliminarily (Figure 3a). Compared with the control plasma of non-administered rats, d-ROMs in the plasma of KBGY-treated rats decreased transiently, showing the minimum level 0.5 h after KBGY administration. In order to confirm the antioxidant effect of KBGY to decrease d-ROMs in the plasma, we repeated experiments by using the plasma prepared 0.5 h after a single administration of KGBY or vehicle (distilled water). As shown in Figure 3b, the level of d-ROMs in the KBGY group was significantly lower compared to that of the vehicle group. This study is the first to demonstrate that KBGY decreases levels of d-ROMs in plasma. The d-ROMs are examined clinically as pathological markers and/or exacerbating factors of inflammation in patients with systemic diseases such as diabetes mellitus, rheumatoid arthritis, sepsis, and fatigue [42,43,44,45]. Our novel findings indicate a potential role for KBGY in the treatment of various diseases mediated by oxidative stress.

### 2.4. Active Constituents that May Contribute to the Antioxidant Activity of KBGY

**Figure 4:**
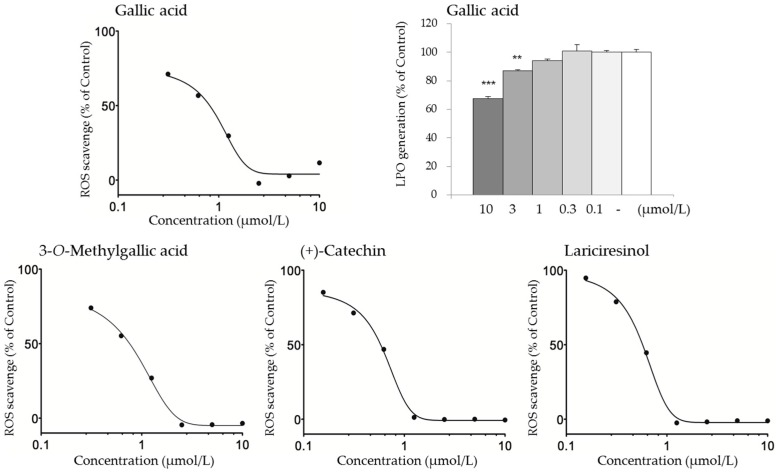
Concentration-dependency of antioxidant KBGY constituents. The active constituents hit in the assays of Table 3 and Table 4 were assayed at the indicated serially-diluted concentrations to assess their repeatability and concentration-dependencies. Data are shown as relative amounts that are percentages of the vehicle control, representing the mean ± S.E. of triplicates (*n* = 3). ** *p* < 0.01, *** *p* < 0.001, significant, respectively, versus vehicle control by Dunnett’s multiple comParison. Statistical analysis revealed that all test samples evaluated in ROS scavenging assays showed significant activities at all concentrations, although star marks indicating significant differences are not represented in the figures.

Because KBGY reduced d-ROMs in vivo, LPO generation assays based on the Fenton reaction using a linoleic acid as a substrate were performed to address active antioxidant constituents of KBGY among the blood-absorbed compounds (Table 3 and Table 4). Evaluation of these 20 compounds indicated that gallic acid, 3-*O*-methylgallic acid, (±)-syringaresinol, lariciresinol, and lyoniresinol exhibited activity at 10 μmol/L which was less than 80% as control percent. Gallic acid significantly inhibited LPO generation at 3 and 10 μmol/L (Figure 4). Next, ROS-scavenging assays were also performed, considering the involvement of ROS for the generation of d-ROMs in plasma. From these assays, gallic acid, 3-*O*-methylgallic acid, (+)-catechin, (±)-syringaresinol, lariciresinol, lyoniresinol, enterodiol, and enterolactone all demonstrated strong anti-ROS activity at 10 μmol/L.

In addition, gallic acid, 3-*O*-methylgallic acid, (+)-catechin, and lariciresinol exhibited concentration-dependent antioxidant effects, showing that the IC_50_ of these compounds were 0.954, 0.744, 0.621 and 0.561 μmol/L respectively.

Gallic acid was quantified at a *C*_max_ of 84.9 ng/mL (equal to 0.499 µmol/L) 0.5 h after administration. Statistically significant activity was observed (71.1% control) at a concentration of 0.313 μmol/L in ROS scavenging assays (Figure 4), while the IC_50_ was 0.954 μmol/L. There was no substantial difference between the *C*_max_ and bioactive concentrations in vitro. KBGY decreased d-ROMs in plasma 0.5 h after oral administration; this time matched the *t*_max_ of gallic acid. This result is of particular interest when discussing active compounds.

## 3. Materials and Methods

### 3.1. Test Sample

KBGY (Lot No. 332034000) and its component crude drugs were supplied by Tsumura & Co. (Tokyo, Japan) in the form of a powdered extract. They were obtained by spray-drying a hot water extract mixture of the following six crude drugs in the ratios provided in parentheses: Cinnamoni Cortex (13.33), Paeoniae Radix (13.33), Persicae Semen (13.33), Poria (13.33), Moutan Cortex (13.33), and Coicis Semen (33.33). As candidate compounds of KBGY, (+)-catechin, (*E*)-cinnamic acid, 5-heneicosylresorcinol, 5-pentadecylresorcinol, 5-tricosylresorcinol, albiflorin, amygdalin, cinnamyl acetate, cinnamyl alcohol, enterolactone, eugenol, gallic acid, methyl cinnamate, pachymic acid, paeoniflorin, paeonimetaboline I, paeonol, tetragalloyl glucose, pentagalloyl glucose, prunasin, resorcinol, and, salicylaldehyde were supplied by Tsumura & Co. with high purities for evaluation by biological tests. The following compounds were purchased from chemical manufacturers: lariciresinol, 2-methoxycinnamaldehyde, 3-*O*-methylgallic acid, 4-*O*-methylgallic acid, adipic acid, azelaic acid, cinnamaldehyde, glutaric acid, oleanolic acid, 3-phenylpropyl acetate, pimelic acid, sebacic acid, suberic acid, and ursolic acid from Wako Pure Chemical Ind. (Osaka, Japan); mandelonitrile from Tokyo Chemical Ind. Co. (Tokyo, Japan); (±)-syringaresinol and lyoniresinol from BioBioPha Co. (Kunming, China); pyrogallol from BIONET (Cornwall, UK); matairesinol from Cayman Chemical (Ann Arbor, MI, USA); dehydropachymic acid, dehydrotumulosic acid, and eburicoic acid from ChemFaces (Wuhan, China); tumulosic acid from Sequoia Research Products (Oxford, UK); and enterodiol, pinoresinol, and secoisolariciresinol from Sigma-Aldrich (St. Louis, MO, USA).

### 3.2. Animals

Male Sprague-Dawley rats were purchased from Charles River Laboratories (Yokohama, Japan). The animals were housed at a temperature of 23 ± 3 °C, with a relative humidity of 50% ± 20% and a 12 h light/dark cycle (lights on from 07:00 to 19:00 h daily). Animals were allowed free access to water and standard laboratory food (MF, Oriental Yeast Co., Tokyo, Japan). After habituation for 1 week, 7–8 week-old rats were used in the present study. This study was approved by and conducted according to the “Guidelines for the Care and Use of Laboratory Animals” of the Laboratory Animal Committee of Tsumura & Co. All surgeries were performed under isoflurane anesthesia, and all efforts were made to minimize suffering.

### 3.3. Measurement of Constituents in KBGY and Its Component Crude Drugs

Preparation of analytical samples was described in our previous report [46]. Briefly, constituents were extracted from 100 mg of each extract powder with 8 mL of methanol/purified water. The extract solution undiluted or diluted 10-, 100-, or 1000-fold with methanol was injected into a LC/MS/MS system and a GC/MS system after pooling with a respective internal standard solution. Dicarboxylic acids (glutaric acid, adipic acid, pimelic acid, suberic acid, azelaic acid, and sebacic acid) were derivatized by reaction with a BF3/butanol solution (10%, *v*/*v*, Sigma-Aldrich) for 1 h at 60 °C. The extract solution was analyzed by GC/MS after hexane extraction. For the preparation of the calibration curve, the same volume of various concentrations of working solution was used instead of the extract solution. Analytical conditions are summarized in Supplementary Materials Tables S1–S4.

### 3.4. Pharmacokinetic Analysis of KBGY Constituents and Associated Metabolites

KBGY prepared in water was administered orally to 16 h fasted rats at a dose of 2 g/10 mL/kg (*n* = 3). Blood was sampled from the abdominal inferior vena cava with a heparinized syringe at 0.25, 0.5, 1, 2, 4, 6, 10 or 24 h after KBGY administration. Plasma was obtained by centrifugation at 1700× *g* for 15 min at 4 °C and stored at −80 °C until analysis.

For quantification of the dicarboxylic acid, 50 µL of standard solution, 100 µL of acetic acid (2%, *v*/*v*), and 650 µL of ethyl acetate were added to 200 µL of plasma samples, followed by mixing and centrifugation (7000× *g*, 5 min). The supernatants were collected and dried with a centrifugal evaporator and concentration system (CC-105; TOMY SEIKO Co., Tokyo, Japan). The residue was dissolved with 100 µL acetonitrile and 50 µL BF3/butanol solution; the dissolved solution was incubated for 1 h at 60 °C. The reaction was terminated by the addition of 50 µL of water and hexane, 150 µL of a saturated solution of sodium chloride, and 400 µL of hexane. The solution was vortexed for 2 min, and the supernatant was obtained by centrifugation (7000× *g*, 5 min). Liquid–liquid extraction was repeated twice. The corrected supernatant was dried with the centrifugal evaporator and concentration system. The dried residue was dissolved in 100 µL of acetonitrile containing 1 µg/mL of *o*-terphenyl (Tokyo Chemical Ind. Co.) as an internal standard (IS). Five microliters of the solution was then injected into the GC/MS system.

For quantification of volatile organic compounds (cinnamaldehyde, methyl cinnamate, 2-methoxycinnamaldehyde, mandelonitrile, salicylaldehyde, eugenol, cinnamyl alcohol, and 3-phenylpropyl acetate), 25 µL of standard solution, 25 µL of 2 µg/mL of 1,4-dibromobenzene (Tokyo Chemical Ind. Co.) as IS, and 500 µL of acetonitrile were added to 200 µL of plasma samples, followed by mixing and centrifugation (7000× *g*, 5 min). Five microliters of the supernatant was then injected into the GC/MS system.

For quantification of other compounds, 25 µL of standard solution, 25 µL of 2 ng/mL of atropine sulfate monohydrate (Wako Pure Chemical Ind.) or niflumic acid (Sigma-Aldrich) as IS, and 750 µL of methanol were added to 200 µL of plasma samples, followed by mixing and centrifugation (7000× *g*, 5 min). The supernatant was obtained and dried with the centrifugal evaporator and concentration system after 150 µL of propylene glycol (1%, *v*/*v*) was added to the supernatant. The dried residue was then dissolved in 60 μL of the HPLC mobile phase for each analytical method, and a 10 or 20 μL portion was injected into the LC/MS/MS system.

Plasma pharmacokinetic data were analyzed by noncompartmental modeling using Phoenix WinNonlin (version 6.3, Certara L.P., St. Louis, MO, USA) to determine various pharmacokinetic constants, including the maximum concentration (*C*_max_), time to maximum concentration (*t*_max_), apparent elimination half-life (*t*_1/2_), and area under the plasma concentration-time curve from zero to last observation time (AUC_0–last_). The *t*_1/2_ was divided by loge^2^/*ke*, where *ke* was the terminal elimination (at least three data points on the descending linear limb) rate constant.

### 3.5. Measurement of d-ROMs in Plasma Samples

Plasma samples were prepared as described above. The levels of d-ROMs in plasma were evaluated using the FREE CARRIO DUO system (Diacron International, Grosseto, Italy) according to the manufacturer’s protocol. In brief, 20 μL of plasma was mixed with an acetic acid buffer (pH 4.8) solution containing ferrous/ferric ions and 20 μL of a chromogen (*N*,*N*-diethyl-paraphenylenediamine), followed by incubation at 37 °C for 5 min. During 3–5 min of incubation, the absorbance at 505 nm was measured. The levels of d-ROMs were calculated automatically and expressed in an arbitrary unit called Carratelli units (U.CARR, 1 unit = 0.8 mg hydrogen peroxide per liter).

### 3.6. Lipid Hydroperoxide Generation Assays

Antioxidant assays using linoleic acid with a purity of 98.0% (Wako Pure Chemical Ind.) were performed using a described protocol [47] with modifications. Briefly, 20 μL of 1% linoleic acid in methanol, 20 μL of 20 mmol/L ferrous chloride in water, 20 μL of test sample in DMSO, and 150 μL of a mixed solution (1:1) of methanol and 20 mmol/L phosphate buffer (pH 7.0) were added to 96-well polypropylene plates. After incubation at room temperature in the dark for 1 h, 10 μL of the reaction products were transferred to 200 μL of a methanol solution containing 39 mmol/L ammonium thiocyanate, 0.2 mmol/L ferrous chloride, and 0.01 mol/L hydrochloride. After incubation for 15 min, absorbance at 500 nm was measured using a microplate reader (Multiskan GO, Thermo Fisher Scientific Inc., Waltham, MA, USA). The concentration of lipid hydroperoxide (LPO) products was calculated conveniently using standard curves of serially-diluted linolein hydroperoxides (Cayman Chemical). The percentage of control was calculated using the following formula; (A − B)/(C − B) × 100, with A = test sample, B = linoleic acid alone, and C = vehicle control. Ascorbic acid was used as a reference agent.

### 3.7. ROS Scavenging Assays

Hydrogen peroxide-dependent oxidation tests were performed using Oxiselect™ In Vitro ROS/RNS Assay kits (Cellbiolab Inc., San Diego, CA, USA) according to the manufacturer’s protocol. Briefly, a test sample was mixed with hydrogen peroxide (final concentration: 2.5 μmol/L) and incubated for 5 min. A fluorescent reaction was started by the addition of a fluorescent probe (dichrolodihydrofluorescin-DiOxyQ) at room temperature. After incubation for 15 min, relative fluorescent units (RFUs) were measured at Em 480 nm/Ex 530 nm using a fluorescent plate reader (Infinite M200; Tecan Trading AG, Männedorf, Switzerland). The percentage of control was calculated using the following formula; (A − B)/(C − B) × 100, with A = test sample, B = fluorescent probe alone, and C = vehicle control. (+)-Ascorbic acid was used as a reference agent.

### 3.8. Statistical Analysis

Results are expressed as means ± S.E., while data of the extracts are shown as single results. Statistical significance was evaluated by one-way analysis of variance (ANOVA), followed by Dunnett’s multiple comParison or unpaired Student’s *t*-test. A probability of less than 0.05 was considered significant.

## 4. Conclusions

There is a growing body of evidence indicating that gallic acid has a wide range of biological activities including antioxidant, anti-inflammation, and antinociception [2]. Gallic acid has been reported to prevent nonsteroidal anti-inflammatory drug-induced gastropathy by blocking oxidative stress and apoptosis [33], and to ameliorate peritonitis-induced sepsis, leading to reduction of changes of redox markers such as LPO and malondialdehyde [32]. Moreover, it has been reported that gallic acid functions as an antagonist of the transient receptor potential ankyrin 1 (TRPA1) channel, and exerts antinociceptive and antiedematogenic effects in various types of dermatitis models induced by TRPA1 agonist, hydrogen peroxide, or carrageenan [34]. The methylation metabolites of gallic acid, 3-*O*-methylgallic acid and 4-*O*-methylgallic acid, have also been reported to be bioactive [35,36,48].

The identification of constituents in KBGY demonstrated that KBGY contains a large amount of gallotannins such as pentagalloyl glucose and tetragalloyl glucose, which originate from Paeoniae Radix and Moutan Cortex [16]. A recent review indicated that pentagalloyl glucose is a very effective and beneficial natural compound in human health, with antioxidant, anti-cancer, anti-diabetic, and anti-inflammatory properties from oral administration [49]. However, previous reports and the results of the present study indicate that gallotannins by themselves are not absorbed to the systemic circulation [49]. Rather, it is supposed that gallotannins can exert actions via enzymatic hydrolysis (mainly microbial tannase) to produce several gallic acids in the gut [50]. Thus, gallotannins may be an important source of gallic acid and its methylation metabolites in plasma.

From the results of ROS-scavenging assays, lignans such as lariciresinol showed strong activity at similar or greater levels as gallic acid, although they were not active at concentrations similar to respective plasma concentrations. Nonetheless, there is a possibility that lignans are involved in the antioxidant activity of KBGY, considering their variation in origins and metabolites [51,52,53]. For instance, enterolactone and enterodiol were immediate metabolites in the body of lariciresinol and (±)-syringaresinol. Lignans possess biological actions as an agonist to estrogen receptor. Although it is necessary to study more, KBGY containing lignans may be beneficial to skin tissue remodeling, because several researchers have demonstrated that estrogen and phytoestrogen promote wound healing [54,55].

Ischemia itself injures tissues, which can lead to the generation of ROS. Especially, the subsequent reperfusion after ischemia follows an oxidative burst, resulting in the production of a large amount of toxic oxygen radicals [56]. Superoxide anion react easily and chemically with NO which expands peripheral blood vessels and increases blood flow, followed by abolishment of the physiological activities of NO and may lead to chronic ischemia [57]. It has been discussed that dietary advanced lipid oxidation end products (ALEs) and advanced glycation end products (AGEs) may be risk factors to human health [58,59]. Therefore, the development of therapeutic strategies for mitigating the inflammatory spiral induced by ROS and improve redox-imbalance systemically is necessary. KBG has been demonstrated to improve microvascular impairment [13,14]; therefore, KBG and KBGY may treat ischemic dermatosis via increasing blood flow in the skin. In fact, long-term administration of KBG has been demonstrated a marked improvement in patients of atopic dermatitis with a high lichenification score [60].

Our study raised the possibility that gallic acid, (+)-catechin, lignans, as well as their derivatives, contribute to the antioxidant effects of KBGY. Gallic acid and (+)-catechin originate from Paeoniae Radix and Moutan Cortex [16,18]. Lignans are mainly derived from Cinnamoni Cortex as shown in Figure 1a. KBG consists of Cinnamoni Cortex, Paeoniae Radix, Persicae Semen, Poria, and Moutan Cortex, and has been reported to decrease concentrations of d-ROMs in a rat model by dietary KBG administration for one month [12]. Our results are consistent with these findings since active antioxidant constituents could be derived from all crude components with the exception of Coicis Semen. In conclusion, our study identified 39 constituents of KBGY, and 23 compounds in the plasma of KBGY-treated rats. The blood pharmacokinetic study revealed that various types of medicinal compounds such as gallic acid, prunasin, paeoniflorin, (+)-catechin, lariciresinol, azelaic acid, and their derivatives, were detected in the plasma. In addition, we verified the antioxidant activity of KBGY for decreasing the levels of reactive oxidative metabolites, which are exacerbating factors of dermatitis, and addressed active constituents among the blood-absorbed compounds. Gallic acid appears to be the most influential constituent to the antioxidant effects of KBGY. The findings of the present study indicate that KBGY may improve redox imbalances induced by oxidative stress in the body, and may be a promising treatment for refractory skin diseases.

## Figures and Tables

**Table 1:** Chemical constituents identified in KBGY extract.

Compound Name	Amount (µg/g KBGY)	Compound Name	Amount (µg/g KBGY)
*Monoterpenoid*		*Flavonoid*	
Paeoniflorin ^a^	17,000	(+)-Catechin ^c^	388
Albiflorin ^a^	5340	*Phenylpropanoid*	
Paeonimetabolin I	275	Cinnamaldehyde ^d^	1260
*Triterpenoid*		(*E*)-Cinnamic acid ^f^	358
Tumulosic acid ^b^	302	Cinnamylacetate ^e^	287
Pachymic acid ^b^	289	Cinnamyl alcohol ^d^	192
Dehydropachymic acid ^b^	91.2	2-Methoxycinnamaldehyde ^e^	70.6
Oleanolic acid and/or Ursolic acid ^a^	31.3	3-Phenylpropyl acetate ^f^	20.5
Dehydrotumulosic acid ^b^	9.76	Methylcinnamate ^f^	5.59
Eburicoic acid ^b^	BQL	Eugenol ^e^	2.18
*Gallotannin*		*Lignan*	
Pentagalloyl glucose ^a^	3190	(±)-Syringaresinol	199
Tetragalloyl glucose ^a^	1260	Pinoresinol	15.0
*Phenol*		Lyoniresinol	7.60
Gallic acid ^a^	2460	Secoisolariciresinol	7.09
Paeonol ^a^	578	Matairesinol	0.217
Salicylaldehyde ^d^	557	Lariciresinol	BQL
3-*O*-Methylgallic acid	110	Enterolactone	BQL
4-*O*-Methylgallic acid	27.4	Enterodiol	BQL
Pyrogallol	9.44	*Dicarboxylic acid*	
Resorcinol	BQL	Azelaic acid	75.2
5-Pentadecylresorcinol	BQL	Glutaric acid	50.8
5-Heneicosylresorcinol	BQL	Suberic acid	47.6
5-Tricosylresorcinol	BQL	Pimelic acid	13.3
*Cyanogenic glycoside*		Adipic acid	12.7
Amygdalin ^g^	9760	Sebacic acid	BQL
Prunasin ^g^	1260		
Mandelonitrile	189		

Methanol/water extracts of dried KBGY (Lot No. 332034000) were analyzed by LC/MS/MS and GC/MS. Amounts of the following compounds were below the quantification limits provided in parentheses (µg/g KBGY); eburicoic acid (16.0), resorcinol (0.800), sebacic acid (40.0), lariciresinol (8.00), enterodiol (8.00), enterolactone (8.00), 5-pentadecylresorcinol (8.00), 5-heneicosylresorcinol (1.60), 5-tricosylresorcinol (8.00), a–g: Each alphabet shows the reference paper or book, [16,17,18,19,20,21,22], respectively.

**Table 2:** Pharmacokinetic parameters of KBGY constituents identified in the plasma of rats treated with KBGY.

Compound Name	*C*_max_ (ng/mL) (μmol/L)	AUC_0–last_ (ng·h/mL)	*t*_max_ (h)	*t*_1/2_ (h)
Poria				
Dehydrotumulosic acid	15.5 (0.032)	39.5	0.5	3.81
Pachymic acid	1.21 (0.002)	5.29	4	-
Tumulosic acid	200 (0.411)	700	0.5	4.66
Paeoniae Radix & Moutan Cortex				
(+)-Catechin	22.6(0.078)	65.1	2	-
3-*O*-Methylgallic acid	12.7 (0.069)	58.6	1	6.59
4-*O*-Methylgallic acid	159 (0.863)	479	1	0.906
Albiflorin	16.1 (0.034)	64.9	0.5	1.6
Gallic acid	84.9 (0.499)	172	0.5	0.772
Paeoniflorin	80.2 (0.167)	299	0.5	2.72
Oleanolic acid and/or ursolic acid	0.927 (0.002)	0.116	0.5	-
Persicae Semen				
Amygdalin	37.7 (0.082)	79.9	0.5	0.657
Manderonitrile	165 (1.239)	251	1	-
Prunasin	1450 (4.910)	3700	1	1.16
Cinnamomi Cortex				
(±)-Syringaresinol	0.213 (0.001)	0.0771	0.25	-
(*E*)-Cinnamic acid	579 (3.908)	916	0.25	23
Cinnamaldehyde	11.5 (0.087)	51.6	1	5.21
Lyoniresinol	0.0408 (0.0001)	0.3	1	11.4
Lariciresinol	1.68 (0.005)	9.84	4	-
Enterodiol	1.03 (0.003)	2.91	0.5	27.3
Enterolactone	1.07 (0.004)	1.33	4	-
Coicis Semen				
Adipic acid	14.4 (0.099)	17.1	6	-
Azelaic acid	54.7 (0.291)	39.9	0.25	-

-: Not calculated.

**Table 3:** Antioxidant activity of blood-absorbed KBGY constituents and their metabolites.

Test Compound	Concentration (μmol/L)	Antioxidant Activity (% of Control)	
		ROS Scavenge	LPO Generation
Gallic acid	10	14.5 ± 0.7	74.0 ± 2.5
3-*O*-methylgallic acid	10	C.I.	77.8 ± 4.3
4-*O*-methylgallic acid	10	86.8 ± 0.2	105.4 ± 2.5
Paeoniflorin	10	100.2 ± 1.4	92.7 ± 0.9
Alibiflorin	10	104.2 ± 1.1	101.6 ± 2.2
(+)-Catechin	10	7.3 ± 1.1	95.4 ± 3.9
Prunasin	10	102.2 ± 1.1	97.9 ± 3.3
Amygdalin	10	106.4 ± 0.5	102.0 ± 2.1
Mandelonitrile	10	99.7 ± 0.6	104.6 ± 1.2
Cinnamaldehyde	10	103.8 ± 1.1	109.8 ± 3.9
(*E*)-Cinnamic acid	10	108.7 ± 2.2	104.3 ± 2.3
Tumulosic acid	10	105.9 ± 2.1	99.4 ± 2.3
Dehydrotumulosic acid	10	106.0 ± 0.4	97.4 ± 3.1
Azelaic acid	10	100.4 ± 0.7	97.9 ± 4.4
Adipic acid	10	97.0 ± 0.9	103.6 ± 2.9
(+)-Ascorbic acid	114	14.5 ± 0.6	27.6 ± 1.7

Test samples quantified at more than 10 ng/mL in the plasma were assessed at a concentration of 10 μmol/L in two assays of hydrogen peroxide-dependent oxidation (ROS scavenge) and lipid hydroperoxide generation (LPO generation). (+)-Ascorbic acid was used as a reference agent. Percentages of control were calculated using the following formula; (A − B)/(C − B) × 100, A: test sample, B: fluorescent probe alone in ROS scavenging assay or linoleic acid alone in LPO generation assay, C: vehicle control. Data are shown as the mean ± S.E. of triplicates. The value of more than 100% exhibits no activity. C.I.: Complete inhibition.

**Table 4:** Antioxidant activity of blood-absorbed lignans derived from KBGY.

Test Compound	Concentration (μmol/L)	Antioxidant Activity (% of Control)	
		ROS Scavenge	LPO Generation
(±)-Syringaresinol	1	39.4 ± 2.4	107.4 ± 4.2
	10	C.I.	68.4 ± 3.3
Lariciresinol	1	C.I.	98.3 ± 1.4
	10	C.I.	79.5 ± 1.9
Lyoniresinol	1	89.3 ± 2.0	96.8 ± 2.9
	10	C.I.	57.4 ± 1.6
Enterodiol	1	67.6 ± 2.4	95.2 ± 4.4
	10	C.I.	84.9 ± 3.1
Enterolactone	1	81.6 ± 2.9	102.1± 2.7
	10	C.I.	86.1 ± 0.9
(+)-Ascorbic acid	114	20.2 ± 0.6	9.9 ± 1.3

Lignans and the metabolites identified in the plasma were assessed at a concentration of 1 and 10 μmol/L in two assays of hydrogen peroxide-dependent oxidation (ROS scavenge) and lipid hydroperoxide generation (LPO generation). (+)-Ascorbic acid was used as a reference agent. Data are shown as the mean ± S.E. of triplicates. C.I.: Complete inhibition.

## Supplementary Materials

The following are available online at: http://www.mdpi.com/1420-3049/22/2/256/s1.
