# Supplementary material for: Oral Administration of the Japanese Traditional Medicine Keishibukuryogan-ka-yokuinin Decreases Reactive Oxygen Metabolites in Rat Plasma: Identification of Chemical Constituents Contributing to Antioxidant Activity
Source: Molecules. 2017 Feb 8;22(2):256. doi: 10.3390/molecules22020256 (PMC6155852; doi:10.3390/molecules22020256)
Supplement: Supplementary file 1 [file molecules-22-00256-s001.pdf]

Supplementary Materials: Oral Administration of the Japanese Traditional Medicine Keishibukuryogan-ka-yokuinin Decreases Reactive Oxygen Metabolites in Rat Plasma: Identification of Chemical Constituents Contributing to Antioxidant Activity

Yosuke Matsubara, Takashi Matsumoto, Kyoji Sekiguchi, Junichi Koseki, Atsushi Kaneko, Takuji Yamaguchi, Yumiko Kurihara, and Hiroyuki Kobayashi

Table S1. LC/MS/MS methods: Ion parameters of test compounds.

Compound Name	Q1Mass (*m*/*z*)	Q3Mass (*m*/*z*)	DP (volts)	CE (volts)	CXP (volts)	HPLC Method ID #
Pachymic acid	546.311	451.4	1	25	30	1
Dehydropachymic acid	544.255	509.3	11	17	14	1
Tumulosic acid	504.257	469.3	11	19	12	1
Dehydrotumulosic acid	502.254	467.3	6	19	14	1
Eburicoic acid	471.253	90.9	166	111	10	1
Ursolic acid	474.317	439.4	61	17	6	1
Oleanolic acid	474.304	439.4	1	15	4	1
Paeonol	167.011	121.1	56	31	16	1
Enterodiol	319.985	267	6	17	16	1
Enterolactone	315.968	299	36	13	18	1
Amygdalin	456.082	323.1	−155	−18	−15	2
Prunasin	293.964	161	−110	−12	−25	2
Paeonimetabolin I	197.033	179.1	−40	−10	−11	2
3-*O*-Methylgallic acid	183.004	168	−5	−18	−17	2
4-*O*-Methylgallic acid	183.003	168.1	−55	−16	−7	2
Gallic acid	169.007	125	−30	−20	−7	2
Pyrogallol	124.904	78.9	−30	−26	−9	2
Resorcinol	108.909	64.9	−60	−18	−9	2
(+)-Catechin	288.949	245	−115	−22	−13	2
Paeoniflorin	479.075	120.9	−5	−36	−51	2
Albiflorin	479.075	120.9	−5	−36	−51	2
Pentagalloyl glucose	938.964	769	−185	−46	−23	2
Tetragalloyl glucose	787.075	617	−140	−38	−29	2
(*E*)-Cinnamic acid	146.911	103	−70	−14	−3	2
Lyoniresinol	438.161	249.1	11	25	16	3
Lariciresinol	378.044	219.1	1	17	6	3
(±)-Syringaresinol	419.123	265	56	11	18	3
Matairesinol	376.019	359.1	6	11	8	3
Pinoresinol	376.014	235.1	1	11	18	3
Secoisolariciresinol	380.044	327.1	1	15	6	3
5-Tricosylresorcinol	431.434	389.5	−185	−50	−19	4
5-Heneicosylresorcinol	403.43	361.4	−190	−48	−19	4
5-Pentadecylresorcinol	319.158	277.3	−135	−38	−17	4
Atropine (IS)	290.019	124.1	111	31	14	1, 3
Niflumic acid (IS)	280.826	236.8	−60	−30	−16	2, 4

HPLC method ID is described in Table S2. Q1: quadrupole 1, Q3: quadrupole 3, DP: declustering potential, CE: collision energy, CXP: collision cell exit potential, IS: Internal standard.

Table S2. LC/MS/MS methods: HPLC conditions.

HPLC Method	HPLC Condition
1	Column: CAPCELL CORE ADME (100 mm × 2.1 mm I.D., 2.7-μm particle size; Shiseido, Tokyo, Japan)<br>Mobile phase (A) 10 mM ammonium acetate, (B) methanol<br>Gradient elution program (% B in A):<br>0–0.5 min, 20%; 0.5–1 min, 20%–40%; 1–4 min, 40%–90%; 4–10 min, 90%–100%; 10–10.01 min, 100%–20%; 10.01–15 min, 20%<br>Other conditions were: flow rate, 0.3 mL/min; column temperature, 40 °C
2	Column: Ascentis Express RP−amide column (100 mm × 2.1 mm I.D., 2.7-μm particle size; Supelco Analytical, Inc., Tokyo, Japan)<br>Mobile phase (A) 0.2 vol % acetic acid, (B) acetonitrile containing 0.2 vol % acetic acid<br>Gradient elution program (% B in A):<br>0–5 min, 5%; 5–11 min, 5%–95%; 11–12 min, 95%; 12–12.01 min, 95%–5%; 12.01–16 min, 5%;<br>Other conditions were: flow rate, 0.3 mL/min; column temperature, 40 °C
3	Column: CAPCELL CORE ADME<br>Mobile phase (A) 10 mM ammonium acetate, (B) methanol<br>Gradient elution program (% B in A):<br>0–1 min, 30%; 1–6 min, 30%–80%; 6–8 min, 80%–90%; 8–8.01 min, 90%–30%; 8.01–13 min, 30%;<br>Other conditions were: flow rate, 0.3 mL/min; column temperature, 40 °C
4	Column: Ascentis Express HILIC HPLC Column (100 mm × 2.1 mm I.D., 2.7-μm particle size; Supelco Analytical, Inc.)<br>Mobile phase (A) 0.2 vol % formic acid, (B) acetonitrile<br>Gradient elution program (% B in A):<br>0–3 min, 95%; 3–8 min, 95%–50%; 8–8.01 min, 50%–95%; 8.01–13 min, 95%<br>Other conditions were: flow rate, 0.2 mL/min; column temperature, 40 °C

LC/MS/MS system: a TripleQuad6500 (AB SCIEX, Tokyo, Japan) equipped with an Agilent 1290 system (Agilent Technologies, Tokyo, Japan).
